# The impact of COVID-19 infection on hip fractures 30-day mortality

**DOI:** 10.1177/1460408620951352

**Published:** 2021-10

**Authors:** Ahmed Fadulelmola, Rob Gregory, Gavin Gordon, Fiona Smith, Andrew Jennings

**Affiliations:** University Hospital of North Durham, County Durham & Darlington NHSF, Newcastle upon Tyne, UK

**Keywords:** Hip fractures, COVID-19, 30-day mortality

## Abstract

**Introduction:**

A novel virus, SARS-CoV-2 has caused a fatal global pandemic which particularly affects the elderly and those with co-morbidities. Hip fractures affect elderly populations, necessitate hospital admissions, and place this group at particular risk from COVID-19 infection. This study investigates the effect of COVID-19 infection on 30-day hip fracture mortality.

**Method:**

Data related to seventy-five adult hip fractures admitted to two units during March and April 2020 was reviewed. The mean age was 83.5 years (range 65-98 years) and most (53, 70.7%) were females. The primary outcome measure was 30-day mortality associated with COVID-19 infection.

**Results:**

The COVID-19 infection rate was 26.7% (20 patients), with a significant difference in the 30-day mortality rate in COVID-19 positive group (10/20, 50%) compared to COVID-19 negative group (4/55, 7.3%), with mean time to death of 19.8 days (95% confidence interval 17.0-22.5). The mean time from admission to surgery was 43.1 hours and 38.3 hours, in COVID-19 positive and COVID-19 negative groups, respectively. All COVID-19 positive patients had shown symptoms of fever and cough, and all ten cases who died were from hypoxia. Seven (35%) cases had radiological lung findings consistent with viral pneumonitis which resulted in mortality (70% of mortality). 30% (n = 6) contracted the COVID-19 infection in the community and 70% (n = 14) developed symptoms after hospital admission.

**Conclusion:**

Hip fractures associated with COVID-19 infection have a high 30-day mortality. COVID-19 testing and chest x-ray for patients presenting with hip fractures, helps in early planning of high-risk surgeries and allows counselling of the patients and family using realistic prognosis.

## Introduction

Recently, a novel virus, SARS-CoV-2 (COVID-19) originating in China at the end of 2019 has been the cause of a fatal global pandemic. At the time of writing, the death toll from COVID-19 infection in the UK has surpassed 30,000 and represents the highest record of national deaths in Europe.^1^ The overall estimates of COVID-19 death rate in the UK is 0.66% increasing to 7.8% in the population of over 80 years of age^2^; higher death rates have been reported in cases with underlying health conditions or those of older age.^3^,^4^ The virus spreads mainly via close contact and respiratory droplets that lead to a high transmission rate in groups in close contact such as hospitalised patients.^5^,^6^

Fragility hip fractures have an estimated incidence of 70,000 cases per year in the UK and are expected to reach 6.3 million world-wide by the year 2050.^7^,^8^ This injury affects elderly populations, necessitates hospital admission, and makes this group particularly prone to COVID-19 infection. Hip fracture itself is associated with increased mortality, and the risk of death tends to cluster in the first 30-days after injury, then decreases thereafter.^9^ The 30-day mortality rate ranges from 3.5%–10%,^10^,^11^ with the National Hip Fracture Database (NHFD) showing a UK 30-day mortality rate of 7%.^12^ Different predictors and risk factors of hip fractures 30-day mortality have been studied,^13^,^14^ however there is no published evidence on the effect of COVID-19 on hip fracture 30-day mortality. The aim of this study is to analyse the effect of COVID-19 infection on hip fracture 30-day mortality.

## Methods

We retrospectively reviewed all adult hip fractures admitted to two units, in March 2020 and April 2020. Patients characteristics, the Nottingham Hip Fracture Score, the Charlson comorbidity index, and place of residence were recorded. COVID-19 was diagnosed according to the national and local guidelines using reverse transcriptase polymerase chain reaction (SARS-CoV-2 RdRp RT-PCR) of throat swap samples. All patients had received low molecular weight heparin (Enoxaparin sodium forty milligrams) daily to prevent thromboembolic events and underwent surgery when deemed safe following multi-disciplinary consultant review by orthopaedics, Care of the Elderly, and anaesthetics.

The primary outcome measure was 30-day mortality of hip fractures associated with COVID-19 infection; secondary outcome measures were, risk factors of 30-day mortality, medical complications, surgical complications, radiological appearance and laboratory results relating to diagnosis and treatment of COVID-19.

The hip fractures 30-day mortality associated with COVID-19 infection was assessed, but due to the small sample size, the analysis was limited to descriptive statistics to avoid Type I error.^15^ Kaplan-Meier test was used to present the trend of Survival and Cumulative hazard between groups. Statistical Package for Social Sciences (SPSS, version 24.0, IBM Co., Armonk, NY, USA) was used.

The study was authorized by the local ethical committee as part of the Clinical Effectivenessand Audit Department (registration number: 852), and was performed in accordance with the ethical standards of the 1964 Declaration of Helsinki as revised in 2000.

## Results

Seventy-five patients presented with hip fractures during the study period (Table 1). The mean age was 83.5 years (range 65–98 years), and 53 were females (70.7%); all hip fractures were due to low velocity injury and three (4%) had associated injuries (one wrist and two humeral fractures). Twenty patients had COVID-19 infection (26.7%).

**Table 1.** Characteristics of patients presented with hip fractures.

	COVID-19 positive patients n = 20	COVID-19 negative patients n = 55
Age (mean)	83.7 years	83.5 years
Gender		
Female	13 (65%)	40 (72.7%)
Mortality		
Yes	10 (50%)	4 (7.3%)
Fracture classification		
Intra-capsular	11 (55%)	39 (71.9%)
Extra-capsular	9 (45%)	16 (29.1%)
Time from admission to surgery (mean)	37.4 hours	39.8 hours
Operations		
Cemented hemiarthroplasty	10 (50%)	36 (65.5%)
Uncemented hemiarthroplasty	1 (5%)	0 (0%)
Dynamic hip screw	6 (30%)	11 (20%)
Intra-medullary nail	2 (10%)	3 (5.5%)
THR	0 (0%)	3 (5.4%)
Conservative	1 (5%)	2 (3.6%)
Type of anaesthesia^a^		
Spinal	8 (40%)	27 (49.1%)
General	11 (55%)	26 (47.3%)
Complications		
Surgical^b^	1 (5%)	0
Medical^c^	0	1 (1.8%)
Nottingham hip fracture score (mean)	6	5.5
Charlson co-morbidity index (mean)	5.4	5.1
Place of residence		
Own home	13 (65%)	38 (69.1%)
Institution	7 (35%)	17 (30.9%)

THR: total hip replacement.

^a^Three patients were managed conservatively and did not need anaesthesia.

^b^Periprosthetic fracture.

^c^Myocardial infarction.

**Figure 1.**
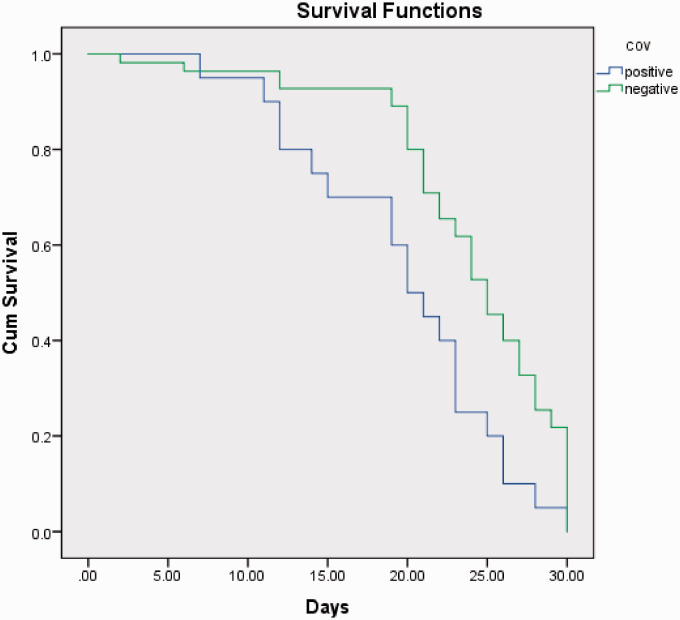
The survivorship of hip fractures patients during the first 30-days post injury related to COVID-19 infection.

There was a significant difference of mortality rate in the COVID-19 positive group (n = 10, 50%) compared to COVID-19 negative group (n = 4, 7.3%), with mean time to death of 19.8 days (95% confidence interval: 17.0–22.5 days, Figure 1). The mean time from admission to surgery was 43.1 hours and 38.3 hours in COVID-19 positive and COVID-19 negative groups, respectively. Six patients (30%) contracted the COVID-19 infection in the community and 14 (70%) developed symptoms after hospital admission 13/14 were tested positive post-operatively with mean time to confirmed infection of 12.6 days.

Two thirds of the cases had intra-capsular hip fracture and cemented hip hemiarthroplasty was the most common operation performed (Table 1); three patients were managed conservatively. The mean Nottingham hip fracture score was 5.6 (range 0–8), and the Charlson comorbidity index had a mean of 5.2 (range 0–9). One patient (1.3%) in the COVID-19 negative group had an acute myocardial infarction post-operatively which was the cause of death. The patient who underwent uncemented hip hemiarthroplasty (1.3%) had a periprosthetic fracture which was managed conservatively. Twenty-four (32%) patients were admitted from their own home and 51 (68%) case came from an institution.

The baseline laboratory results between the two groups are given in (Table 2); c-reactive protein and white cell count were higher in the COVID-19 positive group with a relative lymphopaenia.

**Table 2.** Laboratory findings of patients presented with hip fractures.

	COVID-19 positive patients n = 13	COVID-19 negative patients n = 22
CRP	46.7	33.7
WCC	12.3	11.2
Lymphocytes count	0.7	1.1
Pre-operative haemoglobin g/l	119.1	121.1
Post-operative haemoglobin g/l	100.5	102.1
Haemoglobin drop post-operative g/l	18.4	18.9

CRP: C-reactive protein; WCC: White Cell Count (×10^9^ cells/litre).

**Figure 2.**
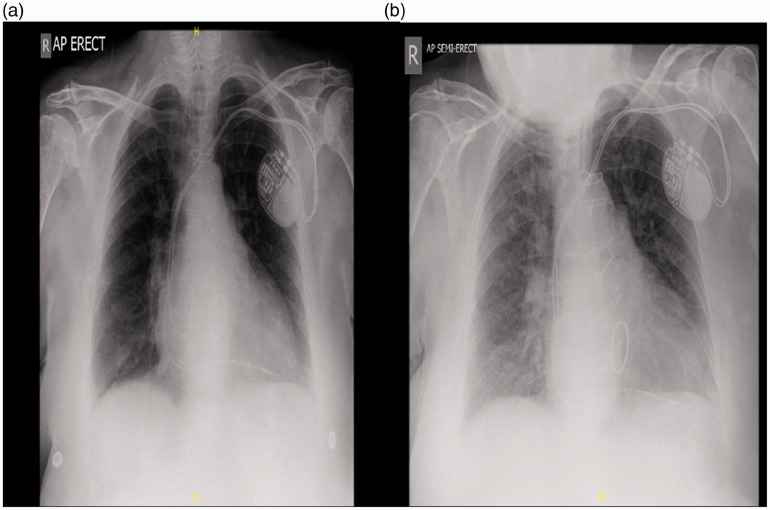
Chest X ray: (a) at admission and (b) Eleven days post index procedure showing features of viral pneumonitis.

All COVID-19 positive cases had shown symptoms of fever and cough and received supplemental oxygen, and all ten who died were hypoxic; all 20 positive patients had undergone chest x-ray or/and chest computerised tomography (CT) to assess lung involvement, which showed seven (35%) cases had radiological lung findings of new pulmonary infiltrates, consistent with viral pneumonitis which resulted in mortality (70% of mortality, Figure 2). None of the ten survivors had features of viral pneumonitis on imaging and 7/20 (35%) received antibacterial therapy.

## Discussion

This study presents the first evidence of high (50%) 30-day mortality in fragility hip fractures associated with COVID-19 infection. Recent estimates based on data from China predict an overall case fatality of 6.4% in those aged over 65 years and 13.4% in over 85 years group.^2^ A population-based study in the UK, including more than 3.8 million subjects, projects an estimate of 4.4% 1-year mortality rate in high risk groups.^16^ Further findings were an expected death rate of 9.7% in over 85 years of age without co-morbidities, which increases to 21.7% in those over 85 years and have more than three co-morbidities. The average time to death from admission was 19.8 days in our study. Similar findings have been reported from the Chinese mainland with mean time from disease onset to death of 17.8 days.^2^,^17^

The COVID-19 virus causes a two-phase immune response.^18^ The second phase, cytokine release syndrome (CRS) which develops after the first week, causes immune-mediated lung damage and/or multi-organ failure, and leads to death. CRS is mediated by leukocytes other than lymphocytes and this leads to leucocytosis associated with lymphopenia. In our study, COVID-19 positive patients had higher mean leukocyte counts and lower mean lymphocytes count compared to COVID-19 negative patients. Various studies have shown that surgery following trauma acts as a ‘‘second hit’’ and induces a strong proinflammatory response.^19^,^20^ We have postulated that in hip fractures associated with COVID-19 infection there are three potential hits, trauma, COVID-19 infection and surgery. Hip fracture surgery may act as a ‘‘third hit’’ that boost the hyper immune state caused by COVID-19 virus which may be a major factor in the increased mortality. A recently published trial involving 6425 patients has shown that the immunosuppressant dexamethasone significantly reduced the death rate in COVID-19 positive patients,^21^ supporting the theory of a hyperimmune reaction leading to higher mortality rates in COVID-19 patients.

We observed radiological evidence of viral pneumonitis in 35% of patients proven to be COVID-19 positive, all of whom died; interestingly, all COVID-19 positive survivors had normal chest x-rays, suggesting that CXR findings of viral pneumonitis could be an indicator of mortality. The main presenting symptoms in our study were cough and fever. Reports from China found that cough, fever and sore throat were the most common presenting feature of COVID-19 infection.^22^,^23^ Mi et al. studied ten patients who died due to COVID-19 associated with orthopaedic fractures, six of them presenting with hip fractures;^24^ 60% of their patients had lymphopenia, similar to our findings.

## Limitations

The limitations of this study include its retrospective nature, relatively small sample size, and potential confounders for mortality. To improve the sample size, data has been collected from two different units. Due to small sample size, we have not used statistical tests as it may result in false positive findings. However, our study reported a clinically important difference in the 30-day mortality between COVID-19 positive and COVID-19 negative hip fractures patients. We have postulated a ‘‘third hit’’ theory, however, further research will be needed to assess the immune response before and after surgery in hip fractures associated with COVID-19 infection.

## Conclusion

On the basis of our study, hip fractures associated with COVID-19 infection have a high 30-day mortality. Whilst we suggest that mortality is greater in the COVID positive group, the numbers are very small in comparison to other recent published works on the topic in which over 6000 patients are included. Our findings may just be chance but we believe they are suggestive of a higher mortality when set against contemporary practices seen in the fight against the virus. Delayed surgery or conservative treatment can be adopted to avoid the ‘‘third hit’’. We have adopted a protocol of COVID-19 testing and CXR, for all patients presenting with hip fractures in our unit. This helps in risk stratification and early planning of high-risk surgeries and allows counselling of the patients and family using realistic prognosis. This study should open a door for nation-wide studies of COVID-19 virus effect on hip fractures.
